# The Search for Natural and Synthetic Inhibitors That Would Complement Antivenoms as Therapeutics for Snakebite Envenoming

**DOI:** 10.3390/toxins13070451

**Published:** 2021-06-29

**Authors:** José María Gutiérrez, Laura-Oana Albulescu, Rachel H. Clare, Nicholas R. Casewell, Tarek Mohamed Abd El-Aziz, Teresa Escalante, Alexandra Rucavado

**Affiliations:** 1 Facultad de Microbiología, Instituto Clodomiro Picado, Universidad de Costa Rica, San José 11501, Costa Rica; teresa.escalante@ucr.ac.cr (T.E.); alexandra.rucavado@ucr.ac.cr (A.R.); 2 Centre for Snakebite Research & Interventions, Liverpool School of Tropical Medicine, Liverpool L3 5QA, UK; Laura-Oana.Albulescu@lstmed.ac.uk (L.-O.A.); rachel.clare@lstmed.ac.uk (R.H.C.); Nicholas.casewell@lstmed.ac.uk (N.R.C.); 3 Zoology Department, Faculty of Science, Minia University, El-Minia 61519, Egypt; mohamedt1@uthscsa.edu; 4 Department of Cellular and Integrative Physiology, University of Texas Health Science Center at San Antonio, San Antonio, TX 78229-3900, USA

**Keywords:** snake venom, antivenom, inhibitors, metalloproteinases, phospholipases A_2_, three finger toxins, peptidomimetic hydroxamates, varespladib

## Abstract

A global strategy, under the coordination of the World Health Organization, is being unfolded to reduce the impact of snakebite envenoming. One of the pillars of this strategy is to ensure safe and effective treatments. The mainstay in the therapy of snakebite envenoming is the administration of animal-derived antivenoms. In addition, new therapeutic options are being explored, including recombinant antibodies and natural and synthetic toxin inhibitors. In this review, snake venom toxins are classified in terms of their abundance and toxicity, and priority actions are being proposed in the search for snake venom metalloproteinase (SVMP), phospholipase A_2_ (PLA_2_), three-finger toxin (3FTx), and serine proteinase (SVSP) inhibitors. Natural inhibitors include compounds isolated from plants, animal sera, and mast cells, whereas synthetic inhibitors comprise a wide range of molecules of a variable chemical nature. Some of the most promising inhibitors, especially SVMP and PLA_2_ inhibitors, have been developed for other diseases and are being repurposed for snakebite envenoming. In addition, the search for drugs aimed at controlling endogenous processes generated in the course of envenoming is being pursued. The present review summarizes some of the most promising developments in this field and discusses issues that need to be considered for the effective translation of this knowledge to improve therapies for tackling snakebite envenoming.

## 1. The Treatment of Snakebite Envenoming: A Challenging Task in Need of Innovation

Snakebite envenomings represent an impactful global public health problem, with the highest incidences occurring in Sub-Saharan Africa, Asia, and Latin America [1,2]. It is estimated that between 1.8 and 2.7 million cases of snakebite envenoming occur annually, resulting in 81,000 to 138,000 fatalities and more than 400,000 people left with permanent sequelae [2]. Snakebite envenoming is a neglected tropical disease [3] since it largely affects impoverished communities with little political voice and contributes to the perpetuation of a vicious cycle of poverty [4,5], hence fulfilling the basic features of these diseases [6,7]. This has prompted international efforts to raise awareness on the impact of this disease and on the need to implement effective strategies to confront it [8,9,10,11]. Consequently, in 2018, the World Health Assembly approved a resolution that urged member states to take action for the prevention and control of snakebites [12], and a global strategy aimed at reducing the number of deaths and disabilities caused by envenomings by 50% by the year 2030 was launched by the World Health Organization (WHO) in 2019 [13].

One of the four pillars of this global strategy is to ‘ensure safe, effective treatment’ [11]. Since the last decade of the 19th century, the mainstay treatment for snakebite envenoming is the timely administration of safe and effective antivenoms [14]. These are preparations of antibodies or antibody fragments generated from the plasma/serum of animals, usually horses, immunized with snake venoms [15,16]. When prepared following Good Manufacturing Practices (GMPs) and with the selection of appropriate venoms for immunization, antivenoms are safe and highly effective in the neutralization of the main manifestations of envenomings, especially those associated with systemic pathophysiological effects [2,15]. However, antivenom treatment poses a series of difficulties that need to be circumvented by novel developments and interventions that could improve and complement antivenoms in the therapy of envenomings. The main limitations of antivenom therapy are:

(1) Antivenoms need to be administered by trained medical and nursing staff in healthcare facilities under conditions that enable the management of potentially serious adverse reactions that may occur following their administration and whose incidence varies depending on the product [17,18]. In settings where long distances exist between the locale of the bite and the nearest healthcare facility, there is a significant delay in receiving antivenom. It has been shown that the time lapse between the bite and accessing medical treatment is strongly associated with the prognosis of the case, as demonstrated in rural settings in Sub-Saharan Africa, Asia, and Latin America, where such delays are common [19,20,21,22].
(2) While antivenoms are usually effective in halting the systemic manifestations of envenoming, such as bleeding, coagulopathies, cardiovascular disturbances, rhabdomyolysis, neuromuscular paralysis, and other systemic effects [2] if administered promptly after the bite, they are less effective in controlling the local manifestations of envenoming. These effects include edema, hemorrhage, myonecrosis, and cutaneous necrosis which develop rapidly around the bite site upon venom injection, as shown in experimental studies [23,24]. Hence, local envenoming can result in permanent tissue damage and disabilities of various types, especially when there is a delay in antivenom administration, generating an expanding wave of social suffering in families and communities [2,25,26].
(3) Antivenoms are relatively expensive products and their availability and accessibility in developing countries is often limited due to various factors [13,27]. In addition, the distribution of antivenoms to rural health posts where most snakebites occur is commonly affected by administrative issues [28]. Antivenoms are often unavailable at the primary health level, hence resulting in people having to travel long distances to be treated in an appropriate setting [21,29,30].
(4) Due to the immunological variation of snake venoms, antivenom specificity has an impact on the treatment of envenoming [31]. Antivenoms are generally effective against the venoms used for immunization and, through cross-neutralization, against venoms of closely related species [15]. However, there are medically relevant venoms for which effective antivenoms are not available in some regions. Furthermore, the immunogenicity of some clinically relevant toxins, such as the low molecular mass three finger toxins (3FTxs), is poor, making it difficult to raise antivenom of high titers against these components [32,33,34].
(5) Liquid antivenoms require a cold chain system for storage and distribution [15], which is often limited in developing countries, hence precluding the distribution of liquid products to rural health facilities in some regions.

Animal-derived antivenoms will remain the centerpiece in the treatment of snakebite envenoming, at least in the short-medium term, and renewed efforts should be fostered towards improving their design and quality, the volume of antivenom made globally available, the procurement and distribution of antivenoms to regions of high snakebite incidence, and the training of medical and nursing personnel in the use of these products along the priorities set by the WHO strategy [13]. In addition, novel approaches aimed at new therapeutic alternatives are being considered. For example, the introduction of recombinant antibody technologies for the development of next generation antivenoms is being pursued and promising experimental results have been reported since different antibody formats can be adapted to specific pharmacokinetic and pharmacodynamic needs [35,36,37].

At the same time, there is an urgent need for novel non-immunological therapies that would complement antivenoms in the management of this disease, as indicated in the WHO strategy [13]. The possibility of introducing therapies that could be administered in the field rapidly after the snakebite, with a good safety profile and without requiring specialized medical personnel, is being actively considered as a step forward in the management of envenomings [38]. An intensive search for safe and effective natural and synthetic toxin inhibitors and other alternative therapies is currently being carried out by many groups at the preclinical level. Some of the most promising advances involve the repurposing of drugs developed by the pharmaceutical industry for other diseases. These drugs have undergone clinical trials and can inhibit key venom components, such as metalloproteinases (SVMPs) and phospholipases A_2_ (PLA_2_s). In addition, the development of novel high-throughput strategies for drug design could be applied to the snakebite field for the development of novel toxin inhibitors. The present review summarizes some of the most promising developments in this field and discusses issues that need to be considered for the effective translation of this knowledge to improve therapies for tackling snakebite envenoming.

## 2. Identifying the Targets: How to Deal with the Complexity of Snake Venom Composition and Actions

Snake venoms are complex secretions which are thought to have evolved in advanced snakes to facilitate prey capture [31,39,40,41], though snakes will also deploy these chemical weapons defensively, as exemplified by human snakebites. Our understanding of the complexity and evolutionary trends of snake venoms has been enriched by means of ‘omic’ technologies, which have provided a growing body of information in the fields of proteomics, transcriptomics, and genomics [40,42]. In addition to contributing to the study of venom evolution, ecology, biochemistry and toxicology, this wealth of data provides valuable insights into the design of more effective antivenoms and of novel snake venom toxin inhibitors [43]. In particular, the combination of proteomics and toxicological analyses, dubbed ‘toxicovenomics’ [41], now allows the identification of the most relevant toxins in particular venoms through the estimation of a Toxicity Score for individual toxins and venom fractions [44]. The assessment of the toxicity of venom fractions should evaluate not only overall toxicity, i.e., lethality, but also other relevant toxic activities of venoms such as hemorrhagic, procoagulant, myotoxic and dermonecrotic activities. The toxic profile to be tested depends on the main clinical effects of a particular venom.

**Figure 1:**
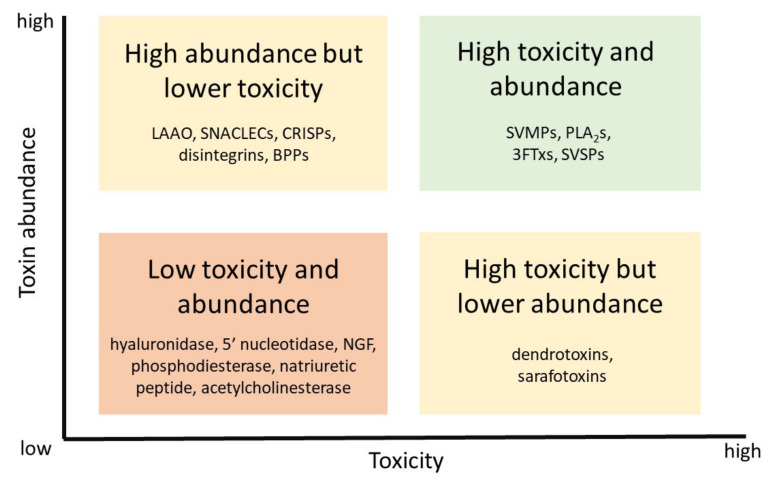
Distribution of snake venom toxins in four groups according to their abundance in venoms and their toxicity. Efforts to develop novel inhibitors should prioritize toxins of high abundance and toxicity, although for some venoms the search of inhibitors of toxins of high toxicity but lower abundance is also recommended. Owing to the intrinsic complexity of venoms, the design of the optimal mixtures of inhibitors should be based on venom composition. Abbreviations: LAAO: L-amino acid oxidases; SNACLECs: C-type lectin-like proteins; CRISPs: Cysteine-rich secretory proteins; BPPs: Bradykinin-potentiating peptides; SVMPs: Snake venom metalloproteinases; PLA_2_s: Phospholipases A_2_; 3FTxs: Three-finger toxins; SVSPs: Snake venom serine proteinases; NGF: Nerve growth factor. See text for details.

The available information facilitates the classification of venom targets into four main categories, based on their toxicity and abundance in medically important snake venoms. These groups include components with: (a) high toxicity and high abundance, (b) high toxicity and lower abundance, (c) high abundance but lower toxicity, and (d) low abundance and low toxicity (Figure 1). In this classification, abundance refers to components which are present in many types of snake venoms, in variable proportion depending on the species. Consequently, we propose that the search for novel snake venom toxin inhibitors should be primarily focused on toxins of high toxicity and abundance (i.e., ‘group a’), and especially on SVMPs, PLA_2_s and three-finger toxins (3FTxs), since they are by far the most abundant toxic components in venoms, with variations between families, and the ones playing key roles in the pathophysiology of most envenomings. The distribution and toxicological profiles of venom components will be discussed next.

### 2.1. Components of High Toxicological Impact and Abundance

#### 2.1.1. Snake Venom Metalloproteinases (SVMPs)

With few exceptions, SVMPs are abundant in viperid venoms, while present in lower amounts in elapid venoms, and in variable proportions in ‘colubrid’ (*sensu lato*) venoms [2,45,46,47]. SVMPs are sub-classified based on their domain composition [48]. Class PI comprises enzymes containing the metalloproteinase domain alone, while class PII includes representatives having a disintegrin domain in addition to the catalytic domain. In turn, class PIII SVMPs contain metalloproteinase, disintegrin-like and cysteine-rich domains. Within each class, there are subclasses encompassing dimeric or truncated variants [49].

SVMPs act on an ample spectrum of substrates, as revealed by proteomic and otheranalyses [50,51]. In terms of their pathophysiological effects, their actions on extracellular matrix components and on coagulation factors determine their ability to induce local and systemic hemorrhage, and coagulopathies, respectively [52]. Other effects induced by SVMPs include inflammation and pain [53], blistering and dermonecrosis [54], myonecrosis [55], and affecting skeletal muscle regeneration [56,57]. All these effects depend on the enzymatic activity of SVMPs, and therefore the search for inhibitors has generally focused on molecules that abrogate enzymatic activity. Thus, SVMPs play a key role in the overall pathology and pathophysiology of envenomings in viperid venoms. The precise role of PIII SVMPs present in elapid venoms is unknown, although they do not seem to contribute to their overall toxicity. ‘Colubrid’ (*sensu lato*) venoms contain PIII SVMPs known to induce hemorrhage and coagulopathy, and which are responsible for the systemic effects characteristic of the scarce severe envenomings caused by this group of snakes [58].

#### 2.1.2. Phospholipases A_2_ (PLA_2_s)

PLA_2_s are ubiquitous components of snake venoms and they display a variety of toxic activities [59]. These enzymes catalyze the hydrolysis of the *sn*-2 ester bond in 1,2 diacyl-3-*sn* glycerophospholipids at the C-2 position by a mechanism dependent on conserved key residues at the catalytic site and the calcium-binding loop [60]. Elapid venom enzymes belong to group I PLA_2_s [61], whereas viperid enzymes are classified as group II PLA_2_s [62]. They share a similar catalytic mechanism but differ in their structure [60]. Many PLA_2_ isoforms, particularly the acidic ones, are devoid of toxicity, whereas other isoforms, most of them cationic, show a diverse set of toxic effects, including neurotoxicity, myotoxicity, nephrotoxicity, edema-forming activity, anticoagulant activity, inhibition of platelet aggregation, hemolytic activity, and cardiovascular effects [59].

In many instances, the toxicity of PLA_2_s is associated with molecular regions different from the catalytic site which enable these enzymes to bind to key targets in cells or coagulation factors in plasma [63]. Upon binding these ‘acceptor’ sites, the disruption of membranes or the inhibition of physiological functions may depend on the hydrolysis of phospholipids, while in other cases their action is based on catalytically independent processes. In addition, a subgroup of PLA_2_s, known as PLA_2_ homologues, have substitutions in key catalytic residues, particularly in Asp 49 which is substituted by Lys and, in some cases, by Arg, Ser, or Asn [64]. These modifications render these isoforms catalytically inactive, while maintaining their toxic profile. The search for venom PLA_2_ inhibitors should aim to address both the inhibition of enzymatic activity (i.e., binding to the active site and the abrogation of the catalytic process), and blockage of the molecular sites that enable these toxins to bind to key targets.

#### 2.1.3. Three Finger Toxins (3FTxs)

3FTxs are abundant components in elapid venoms and in many ‘colubrid’ venoms, but are seemingly absent from viperid venoms [46,47]. 3FTxs are low molecular mass proteins (~6–9 kDa), some displaying a quaternary structure, with a common 3D structure characterized by three loops emerging from a central core and several disulfide bridges [65]. There is a great variety of 3FTxs in snake venoms, which bind to a wide spectrum of targets and exert diverse toxicological effects [66,67]. The best-known representatives are the so-called α-neurotoxins, which have a high affinity for the nicotinic cholinergic receptor located in the motor endplate of skeletal muscle fibers [68], and are responsible for the neuromuscular descending paralysis characteristic of envenomings by many elapid species, including terrestrial elapids and sea snakes [2,69]. Fasciculins are 3FTxs which inhibit acetylcholinesterase [70]. Still, some 3FTxs have evolved to bind to targets not related to the nervous system, such as clotting factors [71].

Another group of 3FTxs, which play an important role in envenomings by some terrestrial elapids of the genus *Naja*, includes the ‘cytotoxins’ or ‘cardiotoxins’. They have the capacity to interact and disorganize cellular membranes, hence leading to plasma membrane damage and cytotoxicity [72,73]. These 3FTxs are the most abundant type of this toxin family found in *Naja* spp. venoms and, particularly in the case of many spitting cobra species, cause local cutaneous necrosis and myonecrosis [24,74]. Local necrosis is generally not well neutralized by antivenoms [24] and often leaves permanent sequelae in the victims [75].

3FTxs constitute a priority toxin group in the search for novel inhibitors because: (a) they are poorly immunogenic, owing to their low molecular mass, and hence complicate the development of high titres of neutralizing antibodies in animals immunized to generate antivenoms. In general, the potency of antivenoms raised against elapid venoms whose toxicity is mostly based on the action of 3FTxs is low, as compared to the potency of viperid antivenoms [76,77,78]. (b) In the case of cytotoxic 3FTxs, in addition to their poor immunogenicity, their effect on tissues upon injection has a rapid onset, hence making treatment with antivenom difficult [24]. This is especially the case when the time lapse between the bite and accessing health centers is delayed, as often occurs in Sub-Saharan Africa and Asia where cobras inflict a substantial number of bites.

#### 2.1.4. Serine Proteinases (SVSPs)

Serine proteinases are widely present in snake venoms and display a variety of activities, some of which exert a role in envenomings, although their contribution is generally less relevant than that of SVMPs, PLA_2_s and 3FTxs. In addition to several activities described in vitro, i.e., kallikrein-like activity, activation of coagulation factor V, protein C and plasminogen, and induction of platelet aggregation [79,80], the most significant actions of SVMPs from a pathophysiological standpoint are the formation of fibrin from fibrinogen (‘thrombin-like’ activity, also known as ‘pseudo-procoagulant activity’) [81,82,83] and prothrombin activation [84,85]. The ‘thrombin-like’ activity results in the defibrinogenation characteristic of many viperid venoms. Despite its name, these SVSPs differ from endogenous thrombin in several ways, and they generate weak fibrin clots that are readily degraded by endogenous plasmin [81].

A relevant group of SVSPs in terms of human envenomings are group C and group D prothrombin activators found in several Australian elapid venoms [85]. These enzymes induce a consumption coagulopathy associated with a drop in the concentration of several clotting factors and defibrinogenation, and contribute to coagulopathies and systemic bleeding described in envenomings by these Australian species [84,86]. Serine proteinases are also involved in cardiovascular alterations associated with increments in vascular permeability through the release of endogenous mediators from plasma proteins [79].

### 2.2. Components of High Toxicity But Lower Abundance

There are families of toxins of high toxicity which are distributed only in the venoms of certain snake species. Thus, they do not have the widespread relevance of the toxins described above but should be considered as targets for the development of inhibitors against envenomings by these species. One example are the dendrotoxins, characteristic of mamba venoms (genus *Dendroaspis*, family Elapidae). These toxins belong to the family of Kunitz-type proteinase inhibitors and act by blocking voltage-dependent potassium channels in neurons [87]. It is likely that some of the unique clinical manifestations of mamba snakebite envenomings [88] are induced by dendrotoxins. Thus, the search for mamba venom inhibitors should consider the inhibition of dendrotoxins.

Another example is the group of sarafotoxins present in the venoms of snakes of the genus *Atractaspis* (family Lamprophiidae, subfamily Atractaspidinae). These toxins are homologues of endogenous endothelins, and exert cardiotoxicity [89]. They play a significant role in the clinical manifestations of *Atractaspis* spp. snakebite envenomings, which include cardiac alterations [88]. Another component that may fit into this category is venom vascular endothelial growth factor (vVEGF). It has been suggested that vVEGF in the venom of *Daboia russelii* could play a role in the development of systemic capillary-leakage syndrome, a severe clinical manifestation in envenomings by this species in Asia [90,91]. Some rattlesnake venoms (genus *Crotalus*) contain low molecular mass myotoxins, a unique type of myotoxic components, which induce muscle contracture and vacuolization by acting on ion channels in skeletal muscle fibers [92], although the role of these toxins in rattlesnake envenoming is not clear.

### 2.3. Components of High Abundance But Lower Toxicity

Snake venoms contain proteins which are widely present in species of the Elapidae, Viperidae and ‘Colubridae’ (*sensu lato*) families, but which do not exert a highly significant role in the overall toxicity of envenomings, although some may play ancillary roles. This is the case of L-amino acid oxidase, for which several functions have been described in addition to its enzymatic activity [93]. However, its role in envenoming has not been proven to be significant. C-type lectin-like proteins (SNACLECs) are also common constituents of snake venoms and have been shown to exert anticoagulant activity in vitro, as well as diverse actions on platelets, such as aggregation, agglutination, and inhibition of aggregation [94,95]. In vivo, the most relevant action described for this group of venom proteins is thrombocytopenia, induced by C-type lectin-like proteins in some viperid venoms which induce von Willebrand factor-dependent platelet aggregation [96,97,98]. This effect contributes to the extent of systemic hemorrhage induced by SVMPs [97,98]. Clinical observations indicate that thrombocytopenia is associated with the frequency of systemic bleeding in envenomings by *Bothrops* species [99,100].

Disintegrins are venom components that bind to cellular integrins and derive from the proteolytic cleavage of PII SVMPs [101]. In vitro, some disintegrins inhibit platelet aggregation, but whether this also operates in the platelet hypoaggregation described for some viperid envenomings remains unknown. Cysteine-rich secretory proteins (CRISPs) are also widely present in snake venoms, although their possible toxic roles have not been demonstrated, despite their several pharmacological activities [102]. Pharmacologically active peptides, such as bradykinin-potentiating peptides (BPPs) are common constituents of viperid venoms, comprising in some cases a relatively high percentage of the venom proteome [46]. Despite their hypotensive action, which paved the way for the development of the potent hypotensive drugs captopril and enalapril [103], their actual role in envenomings remains dubious since injection of fractions rich in BPPs from one venom did not induce evident toxicity in mice [104].

### 2.4. Components of Low Abundance and Low Toxicity

Proteomic studies of snake venoms have identified several components which are present in some venoms at low concentrations, but do not seem to exert a relevant toxic role in envenomings. Examples are 5′ nucleotidase, phosphodiesterase, hyaluronidase, natriuretic peptides, ohanin-like peptides, disintegrin-like and cysteine rich (DC) fragments, acetylcholinesterase, nerve growth factor, and Kazal-type proteinase inhibitors, among others [45,46]. Future studies will uncover whether some of these components exert a role in the overall toxicity of some venoms, but no such evidence currently exists.

## 3. Beyond the Direct Action of Toxins: The Need to Further Understand the Pathophysiology of Envenoming in the Search for Novel Therapies

**Figure 2:**
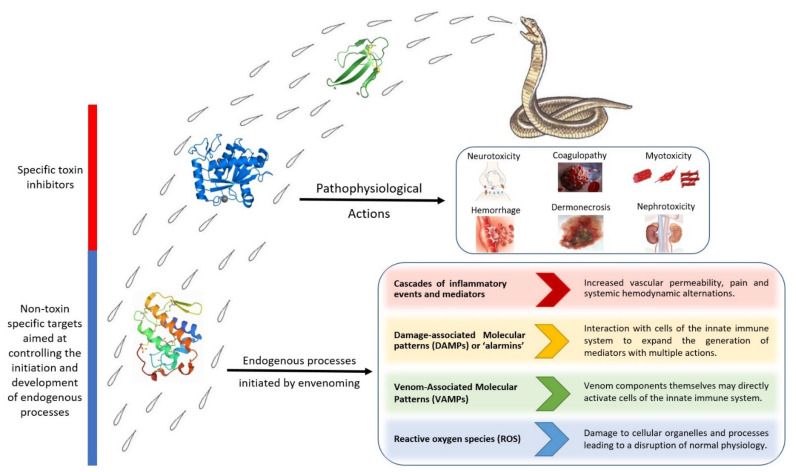
Snakebite envenomings are complex pathophysiological conditions that involve the direct action of venom toxins and the onset of endogenous processes secondary to the actions of toxins. Venoms contain toxins that directly cause neurotoxicity, myotoxicity, dermonecrosis, cardiovascular alterations, coagulopathies, and renal toxicity, among other effects. Concomitantly, the actions of toxins in tissues and cells promote endogenous processes through the action of inflammatory mediators, damage-associated molecular patterns (DAMPs), stimulation of innate immune cells by venom toxins (VAMPs) and the generation of reactive oxygen species (ROS), all of which amplify the action of venoms in a complex interactive scenario. The search for novel drugs for the management of these envenomings should include inhibitors that directly block the action of toxins as well as therapeutics that modulate the endogenous processes in envenomings.

Snakebite envenoming occurs mostly through the direct action of venom toxins in cells, tissues, plasma, and the extracellular matrix, as described. However, envenomings also involve the generation of endogenous processes in the organism that contribute to the pathophysiology of envenomings. For example, the actions of toxins in tissues kick off cascades of inflammatory events and mediators which contribute to an increase in vascular permeability, pain, and systemic hemodynamic alterations [105,106]. Moreover, it has been proposed that the action of venoms on tissues results in the generation of abundant intracellular and extracellular *Damage-Associated Molecular Patterns* (DAMPs) or ‘alarmins’, which interact with cells of the innate immune system to expand the generation of mediators with multiple actions [107,108,109]. The concept of *Venom-Associated Molecular Patterns* (VAMPs) has been proposed, indicating that venom components themselves may directly activate cells of the innate immune system [110]. Understanding these endogenous pathways may pave the way for the design of therapeutic interventions aimed at reducing their deleterious consequences, for example by blocking the receptors or intracellular pathways that mediate the actions of DAMPs and VAMPs. Moreover, since envenomings are associated with the generation of reactive oxygen species (ROS) in the affected tissues, the use of antioxidant interventions has been proposed as an adjunct therapy [111] (Figure 2).

Interventions aimed at reducing the action of venom components, without directly interfering with the toxins, include the use of cholinesterase inhibitors, such as neostigmine, in the case of venoms whose mechanism of action is based on the blockade of the nicotinic acetylcholine receptor (nAChR) by α-neurotoxins, hence increasing the concentration of acetylcholine at the synaptic cleft [112,113,114]. Furthermore, therapeutic interventions may be directed towards improving the regenerative capacity of affected tissues and cells. This is the case of NUCC-390, an agonist of CXCR_4_ receptor, which promotes the regeneration of nerve terminals affected by neurotoxic PLA_2_s that act presynaptically [115], or possible therapies to improve skeletal muscle regeneration after venom-induced myonecrosis [116,117]. Hence, the in depth understanding of venom composition, the mechanisms of action of the most relevant toxins, and the pathophysiology of envenomings will provide valuable information not only for the design of specific toxin inhibitors, but also for other interventions aimed at controlling endogenous pathways playing a role in envenoming.

## 4. The Search for Inhibitors: General Considerations

As indicated above, animal-derived antivenoms are, and will continue to be in the coming years, the mainstay therapeutic for treating snakebite envenoming [2,15]. However, owing to some limitations of these immunobiologicals, alternative venom inhibitors of various origins are being sought as complementary therapies, some of which could be applied in the field rapidly after the onset of envenoming. These novel therapies include recombinant antibodies with high affinity for specific toxins, which can be engineered to provide improved pharmacokinetic profiles and safety, as in the case of humanized antibodies. The topic of recombinant antivenom antibodies has been dealt with in recent reviews [36,118] and will not be considered further here, as the focus of this review is on natural and synthetic inhibitors distinct from antibodies.

A key aspect in the search for novel inhibitory compounds involves testing their inhibitory capacity. The most traditional way to test whether a compound can inhibit the action of venoms or purified toxins is through ‘preincubation-type assays’. These involve the incubation of venom (or toxin) with the inhibitory molecule for a period (generally 30 min) before testing the mixture in the corresponding experimental settings (e.g., both in vitro and in vivo), which vary depending on the effect being evaluated. This is also the method recommended by the WHO for the preclinical in vivo assessment of antivenom efficacy, which is then expressed as the Median Effective Dose (ED_50_), i.e., the ratio venom (or toxin)/antivenom in which the effect of venom (or toxin) is inhibited by 50% [2,15]. A similar method can be used to assess the inhibitory potential of various compounds.

Despite the widespread use of this methodology, it has the caveat that it does not reproduce the actual circumstances of an envenoming, whereby venom is injected first and the treatment is provided at a later stage. Hence, it has been argued that a more appropriate evaluation of inhibitors (and probably also of antivenoms) should be based on ‘rescue assays’, in which the venom is injected first, and the inhibitors are administered at various time intervals after envenoming by using a route of administration that would model the actual route to be used in a clinical case, i.e., intravenous, intramuscular, subcutaneous, or oral [119]. This alternative setting takes into consideration the toxicokinetics of venoms (or toxins) and the pharmacokinetics of the inhibitor and considers possible actions of the inhibitors not necessarily related to direct interaction with toxins. However, it should be noted that such models require further development, as high venom dosing to generate rapid experimental endpoints (i.e., within 24 h) provide considerable challenges to observing therapeutic efficacy in such models. Nonetheless, it is desirable that the search for novel venom or toxin inhibitors should include both types of assays, to gain a more complete understanding of the therapeutic potential of the compound of interest.

Another aspect that has gained attention in recent years is the convenience to search for inhibitory molecules within the arsenal of molecules already developed for other diseases, i.e., those which have gone through preclinical testing assessing their safety and efficacy, or even through phases of clinical testing; indeed, some of these are already being used in a clinical setting in the treatment of other pathologies. This drug repurposing or repositioning strategy will save considerable time and resources during downstream development steps. Thus, in addition to the development of completely novel inhibitors against snake venom toxins, the testing of drugs already developed by pharmaceutical research is a highly promising avenue in the field of snakebite envenomings.

## 5. Inhibitors of Snake Venom Toxins

There is extensive literature on natural and synthetic substances with the capacity to inhibit snake venoms and toxins. The general types of inhibitors will be presented, with a summary of their chemical features and their inhibitory potential. The information presented here is not exhaustive but aims to provide the key aspects of the various types of inhibitors and the evidence supporting their potential for snakebite envenomings.

### 5.1. Inhibitors from Natural Sources

#### 5.1.1. Secondary Metabolites of Plants

Plants have been used for centuries in traditional medicine for treating snakebite envenoming all over the world and constitute a rich source of secondary metabolites that could inhibit venoms and toxins. There is abundant literature on plant-derived extracts or compounds isolated from species from all continents, with the capacity to inhibit specific toxic venom activities [120,121,122,123,124,125]. Most studies have been performed using crude plant extracts, while fewer publications described the isolation, structural characterization, and inhibitory potential of purified compounds. Predominantly, preincubation efficacy protocols have been followed, with fewer studies using rescue assays. Variable results have been obtained when using these different protocols and, generally, efficacy is reduced when extracts or isolated compounds are administered after envenoming [126,127,128]. In some cases, a different protocol has been used based on the administration of the extract before envenoming, simulating a situation in which people ingest the extract prior to going into settings at risk of snakebites [125,129]. Several secondary metabolites with inhibitory activities against venoms or toxins have been identified, including flavonoids, coumestans, phenolic compounds, pterocarpans, alkaloids, steroids, terpenoids, and tannins, among others [120,121,122,123,124]. A detailed analysis of this subject is beyond the scope of this work, but interested readers can consult several reviews [120,121,122,124].

Valuable information can be gathered using an ethnographical approach, based on the knowledge generated in popular medicine in different cultural contexts. This demands close communication between scientists and communities where this knowledge has been developed, conserved, and used [128,130,131]. This rich body of information provided at the community level can bring valuable insights in identifying species of plants, and the specific components of these plants, that hold potential venom-inhibitory compounds.

**Figure 3:**
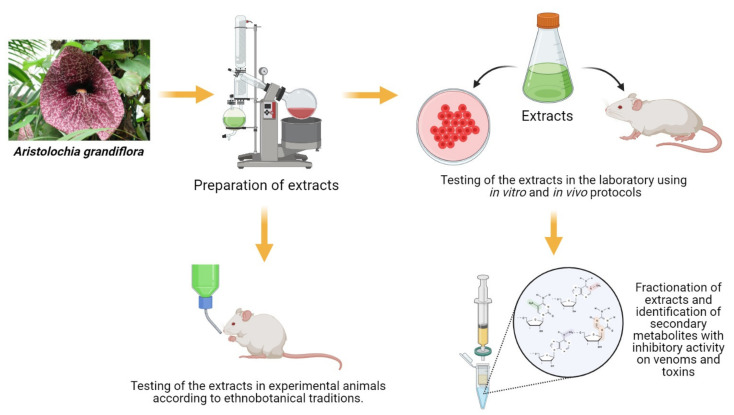
The two main avenues to assess the inhibitory potential of plant extracts and other natural products against the action of snake venoms. Extracts can be tested for their inhibitory action in preclinical models. Extracts that yield positive results can be fractionated and the secondary metabolites responsible for inhibition are characterized and used as lead compounds for further pharmaceutical development. On the other hand, plant extracts can be tested in experimental animal models, on the basis of ethnopharmacological knowledge, by following the way extracts are prepared and administered according to popular traditions. The plant *Aristolochia grandiflora* is a source of molecules with inhibitory potential against various snake venom toxins. Created with BioRender.com.

Thus, there are two main avenues in the study of the inhibitory potential of plants for snakebite envenoming (Figure 3). One involves the replication of practices followed in local communities using plant extracts. The idea is not to purify the inhibitory compounds, but instead to use the whole extracts in the same way that they are prepared in traditional medicine, reproducing in laboratory animals the methods followed in the community for preparing and administering these extracts. The use of whole extracts allows for the detection of synergism between different components. A more advanced stage in this strategy would be to use the extracts in clinical trials in humans, according to traditional practices and following standard bioethical procedures for clinical research. There have been few scientific evaluations of the efficacy of plant extracts in humans [132], an area that requires strengthening, providing that robust preclinical evidence of efficacy and safety has been previously generated. This line of research could validate, in scientific terms, the traditional uses of some plants in the management of snakebite envenoming.

The other avenue in the study of plants as venom antidotes follows the traditional pharmaceutical route, which involves the study of the extract in controlled experimental systems, followed by the isolation and structural characterization of the active principle(s). Such molecules are then chemically modified to improve their efficacy, safety, and pharmacokinetic properties, leading eventually to the development of a new venom inhibitory drug. Several studies have identified and characterized active principles from plants which inhibit various types of toxins. In few instances, the structural characterization of the inhibitor-toxin complex has been described via crystallography, as in the case of aristolochic acid [133], rosemarinic acid [134], and caffeic acid [135]. Moreover, docking and molecular dynamics simulations have also been used to analyze the interaction of plant-derived metabolites with snake venom PLA_2_s and SVMPs [136,137,138]. Despite abundant research in the field of plant extracts and snake venoms, to the best of our knowledge no venom-inhibitory drug derived from a plant secondary metabolite has been approved so far for clinical use in the treatment of snakebite envenomings.

#### 5.1.2. Animal Plasma Proteins

Protein inhibitors of SVMPs and venom PLA_2_s are present in the plasma of several animal species and constitute an innate immune mechanism to resist the action of venoms [139]. SVMP inhibitors belong to several families of proteins, such as the cystatin superfamily (in the plasma of snakes), the immunoglobulin superfamily (in the plasma of opossums and other species), the ficolin/opsonin P35 family (in the plasma of a hedgehog), and other types of inhibitors from snakes and rodents [139,140]. Their inhibitory capacity is generally based on the formation of non-covalent complexes with SVMPs, although the precise structural determinants responsible for binding have not been elucidated.

The PLA_2_ inhibitors present in the plasma of snakes have been classified into three groups: (α) proteins with a C-type lectin-like structure, including a carbohydrate recognition domain, (β) proteins with leucine-rich repeats, and (γ) proteins with a three-finger motif [141]. All form non-covalent complexes with venom PLA_2_s. A PLA_2_ inhibitor belonging to the immunoglobulin superfamily is present in the plasma of an opossum species. It inhibits the myotoxic and cytotoxic activities of a myotoxic PLA_2_ and PLA_2_ homologue from *B. asper* venom, while not inhibiting the catalytic activity of an Asp49 myotoxic PLA_2_ [142]. As with SVMP inhibitors, the structural details of PLA_2_ inhibition by these proteins remains largely unknown. The identification of the molecular regions responsible for enzyme binding and inhibition may pave the way for the synthesis of inhibitory peptides that could eventually be useful in the treatment of envenomings by abrogating the action of these two types of venom components.

#### 5.1.3. Mast Cells and Their Products

It has been demonstrated that mast cells constitute an innate immune mechanism to counteract the toxicity of venoms produced by various animals, including snakes [143,144]. This is based on the action of mast cell proteinases, especially tryptase, which selectively cleaves venom toxins [145]. In addition, heparin, another mast cell-derived substance was shown to inhibit myotoxic and cytotoxic toxins in viperid venoms [146,147]. It was proposed that human recombinant tryptase β could be used to treat snakebite envenomings by administering it at the site of venom injection, hence detoxifying venom components in situ. The potential use of tryptase β and of heparin in snakebite envenoming awaits further experimental and clinical studies.

### 5.2. Synthetic Inhibitors

#### 5.2.1. Metalloproteinase Inhibitors

Endogenous zinc-dependent metalloproteinases belonging to the superfamily of metzincins (MMPs, ADAMs and ADAMTs) play a wide variety of physiological roles and are involved in several pathophysiological conditions, including cancer, arthritis and other inflammatory conditions, pulmonary disease, and sepsis [148,149,150,151]. Thus, an intensive search for inhibitors of endogenous metalloproteinases has taken place during the past few decades. The first inhibitors of MMPs were based on the combination of a peptidomimetic moiety, resembling the sequence of natural substrates of these enzymes, and a zinc-binding motif which, initially, was based on hydroxamate groups which interact with the zinc in a bidentate model [152]. These first-generation inhibitors, such as batimastat and marimastat, showed Median Inhibitory Concentrations (IC_50_) values in the nanomolar range and were considered promising candidates for clinical trials. The results of these trials, however, were disappointing, mostly because of poor bioavailability, lack of specificity for selective MMPs and, consequently, the occurrence of side effects [153,154]. These early failures led to the development of novel inhibitors of higher selectivity to specific MMPs, mostly based on the synthesis of groups adapted to the geometry of the variable S1ʹ pocket of different MMPs, and on the use of chelating moieties with lower zinc affinity [152]. An alternative approach has involved the search for inhibitors that block exosites in the hemopexin-like domain of MMPs instead of acting on the catalytic site [155].

Owing to the structural similarity in the catalytic site between SVMPs and MMPs, both members of the metzincin superfamily, a ‘piggy-back’ approach has been implemented in the field of snakebite in search for SVMP inhibitors, taking advantage of the enormous body of work done in the field of MMP inhibitors. Interestingly, the issues of low specificity and side effects described for some MMP inhibitors do not represent a problem in the field of SVMP inhibition because (a) having low specificity would guarantee inhibition of SVMPs in different venoms with different structural features at the S1ʹ site, and (b) the side effects described in clinical trials result from the repeated administration of inhibitors over long time periods required for the treatment of chronic conditions, whereas in the case of snakebite envenoming, administration (whether single or multiple dosing) would likely be restricted to the first 24–48 h post-envenoming due to the acute nature of the clinical manifestations of this neglected tropical disease. Therefore, it is expected that, in the application of metalloproteinase inhibitors to SVMPs, lack of specificity would be desirable. Indeed, some of the first generation MMP inhibitors, such as batimastat and marimastat, have yielded positive results in preclinical models of envenoming following the administration of viperid venoms and purified SVMPs [156,157,158,159]. Marimastat has the advantage over batimastat in that it has a better bioavailability profile owing to its solubility, which allows for oral administration [152].

**Figure 4:**
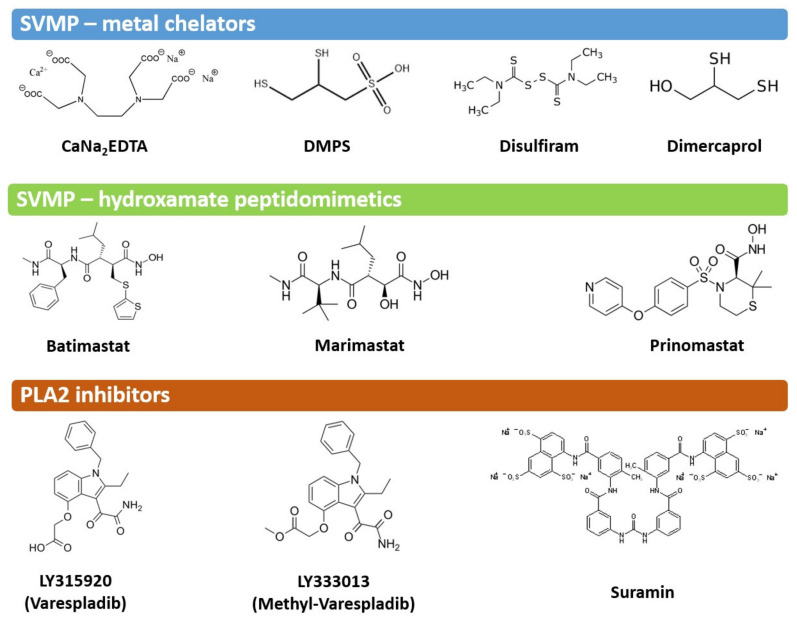
Structures of some of the synthetic compounds that have shown inhibitory activity against snake venoms toxins. Inhibitors of SVMPs include chelating agents, which work by removing the zinc atom of the catalytic site of these enzymes, and hydroxamate peptidomimetics which, in addition to having a chelating moiety (hydroxamate), contain a peptidic sequence that fits within the catalytic region of these enzymes. PLA_2_ inhibitors include Varespladib and its methyl-derivative, and the antiparasitic drug suramin.

The possible use of chelating agents, in clinical use for the treatment of metal intoxication, such as CaNa_2_EDTA, 2,3-dimercapto-1-propanesulfonic acid (DMPS), dimercaprol (British anti-Lewisite), and tetraethyl thiuram disulfide (Disulfiram), have also been assessed in experimental animal models of envenoming, with positive results [157,160,161,162]. However, because these molecules essentially work by chelating the zinc ion, without having a moiety that interacts with residues at the catalytic site, their in vitro SVMP inhibitory potency is lower when compared to peptidomimetic inhibitors [157,159]. Despite this observation, the metal chelator DMPS exhibited impressive in vivo neutralization of the local and systemic effects of SVMP-rich venom from the saw-scaled viper *Echis ocellatus*, including outperforming marimastat in a pre-incubation model of envenoming, and providing protection against lethality when delivered post-venom delivery, including when dosed orally [159,161]. These findings, coupled with DMPS already being a licensed medicine and exhibiting good oral bioavailability, make it a strong candidate for transition into clinical trials for the treatment of snakebite. Other chelating agents that have shown efficacy against SVMPs in pre-incubation experiments are the biphosphonate clodronate and the tetracyclyne doxycycline [163]. Figure 4 depicts the structures of some of the SVMP inhibitors that have been preclinically tested. A novel approach for generating SVMP inhibitors is based on the engineering of a biomimetic of endogenous tissue inhibitors of metalloproteinases (TIMPs) by introducing three binding elements in a synthetic tetrapolymer [164]. An unexplored but interesting possibility is the search for molecules that could interfere with exosites in SVMPs, particularly in the case of PIII SVMPs which exert systemic effects. However, this possibility awaits for the identification of exosites in the disintegrin-like and cysteine-rich domains of these SVMPs.

#### 5.2.2. Phospholipase A_2_ Inhibitors

The PLA_2_s constitute a large superfamily of enzymes classified within several groups and subgroups that belong to six main types [165]. One of these groups includes secretory PLA_2_s, which comprises a large collection of enzymes of 14–18 kDa containing 6–8 disulfide bonds. Secretory PLA_2_s belong to ten different groups which play relevant roles in physiological and pathophysiological processes [165]. Group IB PLA_2_s are present in pancreatic secretions and have a digestive role, whereas group IIA PLA_2_s are expressed in high concentrations in inflammatory exudates and have been associated with diverse disease conditions such as cancer, arthritis, atherosclerosis, and cardiovascular disease [166]. The inflammatory role of group IIA PLA_2_s is related to their ability to cleave arachidonic acid in phospholipids, which is then used in biosynthetic pathways to generate eicosanoids, i.e., prostaglandins, leukotrienes and thromboxanes which exert multiple inflammatory actions [167].

Secretory PLA_2_s are abundant in snake venoms, often comprising a significant portion of their proteomes [2,45]. As in the case of metalloproteinases, the similar catalytic mechanism of human and venom PLA_2_s allows for the possibility of using inhibitors developed for human enzymes as potential inhibitors of venom enzymes. University and industrial laboratories have searched for potent PLA_2_ inhibitors, aimed at treating clinical conditions where PLA_2_s, especially inflammatory group IIA PLA_2_s, play a key role in pathophysiology. Examples of such synthetic inhibitors include sulfonamides, amides, oxadiazolones and indoles [165].

The most promising inhibitors of human group IIA PLA_2_s are the indoles LY315920 (Varespladib) and its prodrug LY333013 (methyl Varespladib) [166,168]. These drugs have been tested in clinical trials for rheumatoid arthritis, sepsis, and cardiovascular disease [169,170,171]. Although, for several reasons, the clinical use of these drugs was abandoned following phase II and phase III clinical trials, recent studies suggest that they can be repurposed for inhibiting venom PLA_2_s. Lewin et al. [172] demonstrated that LY315920 exhibits broad spectrum inhibition of PLA_2_ activity in many viperid and elapid snake venoms. Rescue-type experiments evidenced the ability of LY315920 and LY333013 to prevent or delay death in mice and piglets envenomed with lethal doses of potent neurotoxic elapid venoms whose mechanism of toxicity is based on the presynaptic action of neurotoxic PLA_2_s [173,174,175]. LY315920 was able to prevent the development of the neuromuscular blockade induced by *Oxyuranus scutellatus* venom in nerve-muscle preparations ex vivo. When the inhibitor was added to the preparation after venom, it was effective at abrogating venom-induced blockade at times when antivenom is ineffective [176].

LY315920 was also effective in inhibiting the cytotoxic effect of the venom on C2C12 myotubes and reducing local myonecrosis induced by crude venoms and myotoxic PLA_2_s from the venoms of two viperids and one elapid, even when administered after venom injection [177]. Moreover, this inhibitor also reduced the extent of local tissue damage and improved muscle regeneration in experimental envenomings by *Deinagkistrodon acutus* [178]. PLA_2_s exert anticoagulant activities owing to the hydrolysis of phospholipids which are required for blood clotting or by directly binding to some clotting factors [179]. LY315920 has been shown to inhibit the anticoagulant activity of a variety of snake venoms by inhibiting PLA_2_ activity [180,181,182,183].

In addition to catalytically active Asp49 PLA_2_s, viperid venoms contain PLA_2_ homologues characterized by substitutions at residue 49 (Asp substituted by Lys or other residues) and in the calcium binding loop, which render these proteins enzymatically inactive [64]. Despite their lack of catalytic activity, these homologues are myotoxic, and induce pain and inflammation [184,185,186]. Interestingly, LY315920 partially inhibits myotoxicity in vivo and cytotoxicity on C2C12 myoblasts in culture induced by these homologues, suggesting that this inhibitor is also effective against these isoforms. The structural analysis of the binding of LY315920 to a Lys49 PLA_2_ homologue indicates that it fits into the hydrophobic channel and binds to the His48 and Lys49 residues. In addition, it also prevents the binding of the ‘membrane disrupting site’ of this myotoxin to its membrane target [187]. The glycosaminoglycan heparin inhibits the action of a myotoxic PLA_2_s homologue by interacting with cationic residues of the C- terminus, which are involved in the membrane-destabilizing activity of this toxin [188]. Suramin, a polysulphonated synthetic compound used in the therapy of parasitic diseases, has also been shown to inhibit the myotoxic and cytotoxic activities of Lys49 PLA_2_ homologues, and the structural basis of the interaction of these myotoxins with suramin has also previously been investigated [189,190,191]. Figure 4 depicts the structures of some PLA_2_ inhibitors that have been tested against snake venoms in preclinical studies.

#### 5.2.3. Serine Proteinase Inhibitors

Although serine proteinases are less relevant, as compared to SVMPs and PLA_2_s, in the overall toxicity of snake venoms, in some cases they display relevant toxic activities that should be counteracted in the treatment of envenomings. The search for venom serine proteinase inhibitors has received much lower attention than that for SVMP and PLA_2_ inhibitors. As in the former case, the most effective strategy is the repurposing of drugs which inhibit these enzymes that have been developed for treating other pathologies.

One of the few studies involving snake venoms using a serine proteinase inhibitor approved for human use, nafamostat, showed that, despite inhibition of SVSP activity in vitro, it did not provide protection against the lethal effects of *Echis ocellatus* venom, and that its addition to a mixture of SVMP and PLA_2_ inhibitors did not improve the efficacy of the mixture in neutralizing the venom toxicity of five viperid species [159]. It remains to be determined whether serine proteinase inhibitors could be effective in the treatment of coagulopathies induced by venoms whose procoagulant activity is predominantly based on the activity of serine proteinases, such as some Australian elapids, and viperids like *Crotalus durissus* and *Lachesis* spp. [84,192,193]. One issue to consider when using serine proteinase inhibitors designed to inhibit human enzymes is the potential side effects of such inhibitors, especially regarding their anticoagulant effects in the context of envenomings characterized by coagulopathies. Thus, in contrast to the generic inhibitors proposed above for SVMP and PLA_2_ toxins, the design of inhibitors with specificity towards SVSP venom enzymes is recommended to avoid undesirable off target effects.

#### 5.2.4. Three Finger Toxin Inhibitors

One of the greatest challenges in the design of inhibitors for the therapy of snakebite envenomings relates to the inhibition of 3FTxs, which are particularly abundant in elapid venoms and responsible for neurotoxicity (i.e., neuromuscular paralysis) and cytotoxicity (i.e., tissue damage). The strategy of repurposing drugs developed for other human diseases, quite useful in the case of SVMP and PLA_2_ inhibition, does not apply in the case of 3FTxs due to their unrelatedness to human drug targets and, therefore, novel drugs need to be designed to attain this goal. Fortunately, there are methodological platforms that allow the development of potentially effective inhibitors. A few possibilities are discussed next.

Post-synaptically acting α-neurotoxins of the 3FTx family bind with high affinity to nAChRs at the motor endplate of skeletal muscle fibers, inducing a blockade in neuromuscular transmission and a life-threatening flaccid paralysis [194]. A decoy receptor approach, using mimotopes synthesized based on recombinant humanized nAChR subunits, has been proposed and preliminarily explored [195]. The binding of such mimotopes to neurotoxins would preclude their interaction with native receptors, hence abrogating their effect. One issue with this approach is that different α-neurotoxins may bind to different sequences in the receptor. This could be circumvented by first identifying the most relevant α-neurotoxins in a variety of venoms, through a toxicovenomic approach, and then recognizing the sequences or mimotopes of the receptor that bind these toxins. A high throughput methodology has been applied to address this issue, i.e., label-free bio-layer interferometry, which enables the study of the binding of different venoms and neurotoxins to various molecular regions of the receptor [196]. This has allowed the recognition of orthosteric and allosteric sites in the receptor responsible for neurotoxin binding [197]. The identification of these sites may lead to the synthesis of a diverse set of mimotopes that could bind and block a wide variety of α-neurotoxins.

Another avenue for the design of 3FTx inhibitors is based on aptamers, i.e., short single stranded DNA or RNA oligonucleotides which show variable three-dimensional shapes and therefore present high versatility for binding different targets, including toxins [198,199]. High throughput methods have been implemented for the design and screening of aptamers that bind 3FTxs, such as α-bungarotoxin from *Bungarus multicinctus* venom [200]. Aptamers designed to bind α-bungarotoxin also interact with cytotoxins (cardiotoxins), other 3FTxs present in elapid venoms which cause tissue damage. These aptamers inhibited the membrane-disrupting activity of a cytotoxin [201]. Thus, the development of aptamers, ideally a mixture of oligonucleotides that could bind and inhibit a variety of 3FTxs, must be pursued as a therapeutic alternative to counteract the toxicity of these venom components. Similarly, high throughput screening of synthetic peptides is another alternative for the discovery of molecules that bind and block the action of 3FTxs [202].

**Figure 5:**
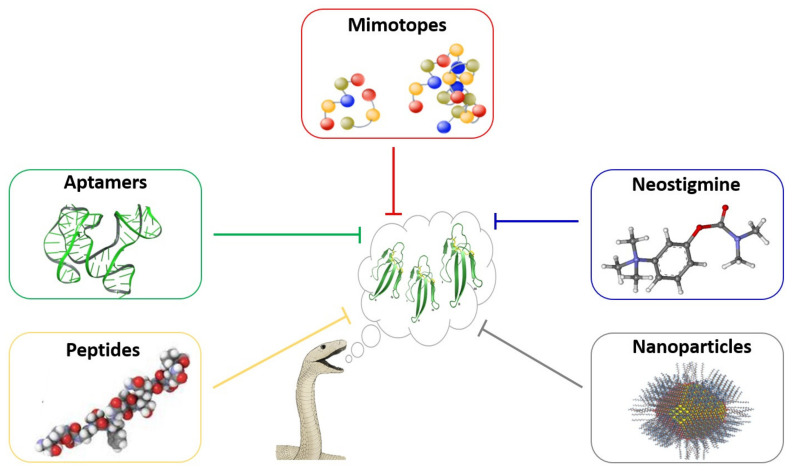
Possible sources of inhibitors against the family of 3FTxs, abundant in elapid snake venoms and responsible for neurotoxicity and cytotoxicity. Aptamers, peptides, and nanoparticles can be designed as to bind with high affinity to these toxins. Likewise, mimotopes, with sequences of the receptors of these toxins, especially the nicotinic acetylcholine receptor, could block toxins’ interacting sites with receptors. Neostigmine, albeit not being an inhibitor of neurotoxins, inhibits acetylcholinesterase at the neuro-muscular junction, thus increasing the local concentration of the neurotransmitter without directly blocking the toxin.

Another promising strategy in the search for 3FTx inhibitors relies on the design of synthetic nanoparticles with the ability to bind and inhibit specific venom components [203]. Abiotic synthetic hydrogel nanoparticles were designed through an iterative process of ‘directed chemical evolution’ and showed a broad spectrum of interaction with 3FTxs and PLA_2_s from a variety of elapid venoms [204]. These nanoparticles were effective at inhibiting the cytotoxic effect of the necrotizing cobra venoms of *Naja nigricollis* and *N. mossambica* on a myogenic cell line and inhibited the dermonecrotic activity of *N. nigricollis* venom in mice. This inhibition was observed not only in experiments where nanoparticles and venom were incubated prior to injection, but also when nanoparticles were administered after venom injection [204]. Owing to the versatility of the interactions between nanoparticles and proteins, they constitute a promising alternative for the development of potent inhibitors of neurotoxic and cytotoxic 3FTxs, and of other toxins as well. Figure 5 summarizes the various options being explored to develop inhibitors of 3FTxs.

#### 5.2.5. Inhibitors of Other Types of Toxins

Depending on the venom, other types of enzymes and toxins may exert a role in envenoming and, therefore, inhibitors against these components may also warrant some attention as secondary targets after those described above. For example, hyaluronidase is a venom component which plays a role as a spreading factor through the degradation of hyaluronic acid in the matrix and is possibly involved in local tissue damage [205]. Several inhibitors of venom hyaluronidase have been described, such as aristolochic acid, alkaloids, flavonoids, sodium chromoglycate, and polysaccharides, among others [205,206]. Sodium chromoglycate and sodium auro-thiomalate were effective in reducing the local and systemic toxicity of two snake venoms [206]. Efforts have been also directed towards the search of LAAO inhibitors by repurposing FDA-approved drugs [207]. Moreover, the search for inhibitors against other non-enzymatic toxins, such as disintegrins, C-type lectin-like proteins and venom vascular endothelial growth factor, which exert toxic activities in some venoms, should be pursued.

## 6. The Potential of Photobiomodulation Therapy for Snakebite Envenoming

Photobiomodulation is a type of phototherapy that includes low-level laser (LLL) and light emiting diode (LED); it utilizes light wavelengths between 600 and 1000 nm to deliver radiation to tissues. It is a non-invasive form of phototherapy used in a variety of musculoskeletal diseases [208]. This therapeutic option has been explored at the experimental level for the treatment of local pathology induced by *Bothrops* spp. snake venoms (see review by Silva et al., 2018 [209]). These studies have demonstrated that these therapies, when applied after venom injection in animals, reduced venom-induced local effects such as edema [210,211], hyperalgesia [211,212], hemorrhage [213], and myonecrosis [211,214,215].

Despite these promising results, the mechanisms by which these therapies reduce venom toxicity in vivo are not clear at present. Evidence indicates that these do not depend on a direct effect of the light on the venom, since the irradiation of venoms before injection did not affect toxicity [211,215]. Therefore, the inhibitory effect is based on the action of light on tissue components, rendering them less susceptible to the venoms. When tested on a myoblast/myotube cell culture, LLL reduced snake venom cytotoxicity and increased myoblast differentiation into myotubes [216]. The mechanisms of inhibition of venom-induced hyperalgesia have been also investigated [217]. Photobiomodulation reversed venom-induced mechanical hyperalgesia and allodynia and decreased venom-induced Fos expression, as well as the mRNA levels of IL-6, TNF-α and B1 and B2 kinin receptors. Since these forms of therapy are already in use in the clinic for several conditions, the possibility of designing and implementing clinical trials in envenomings by snakes that inflict significant local tissue damage would be a relevant task, and indeed such trials are being planned in Brazil (Stella R. Zamuner, personal communication).

## 7. Towards the Introduction of Venom Inhibitors for Clinical Use: Priority Tasks in Preclinical Testing

The search for novel natural and synthetic inhibitors of venom toxins, and the analysis of the inhibitory potential of known inhibitors, should continue and new options need to be explored. At the same time, priority actions are recommended for the repurposing of inhibitors that have already gone through phases of clinical testing for other pathologies, such as inhibitors of SVMPs (e.g., hydroxamate peptidomimetics, metal chelators) and PLA_2_s (e.g., Varespladib-LY315920). These are the most likely candidates to go through intensive preclinical and, eventually, clinical trials in snakebite envenomings. Previous studies with DMPS, batimastat and marimastat or LY315920 have demonstrated their efficacy for reducing the toxicity of SVMP-rich and PLA_2_-rich venoms, respectively [157,158,159,161,175]. Furthermore, the combined use of SVMP and PLA_2_ inhibitors in a dosing regimen provided increased therapeutic efficacy than each inhibitor alone and prevented death in mice receiving lethal doses of five medically important viperid venoms [159], hence suggesting that this combination is likely to have broad inhibitory potential for viperid venoms.

While such an approach is rational, as most viper venoms contain numerous SVMP and PLA_2_ toxin isoforms in abundant yet varying amounts, selection of the most appropriate inhibitor combination will require careful consideration, in terms of optimization of dosing and therapeutic potency, stable formulation of the distinct inhibitory components, and ensuring freedom from drug-drug interactions [38]. There are cases of medically relevant snake venoms whose inhibition may require the use of other substances in addition to SVMP and PLA_2_ inhibitors. This demands a careful case-by-case analysis of the venoms to be inhibited. Ultimately, the selection of new snakebite inhibitors will initially require robust evidence of preclinical efficacy against envenoming, and thus some recommendations relating to the design of such studies are outlined below.

### 7.1. The Design of Preclinical Experiments

**Figure 6:**
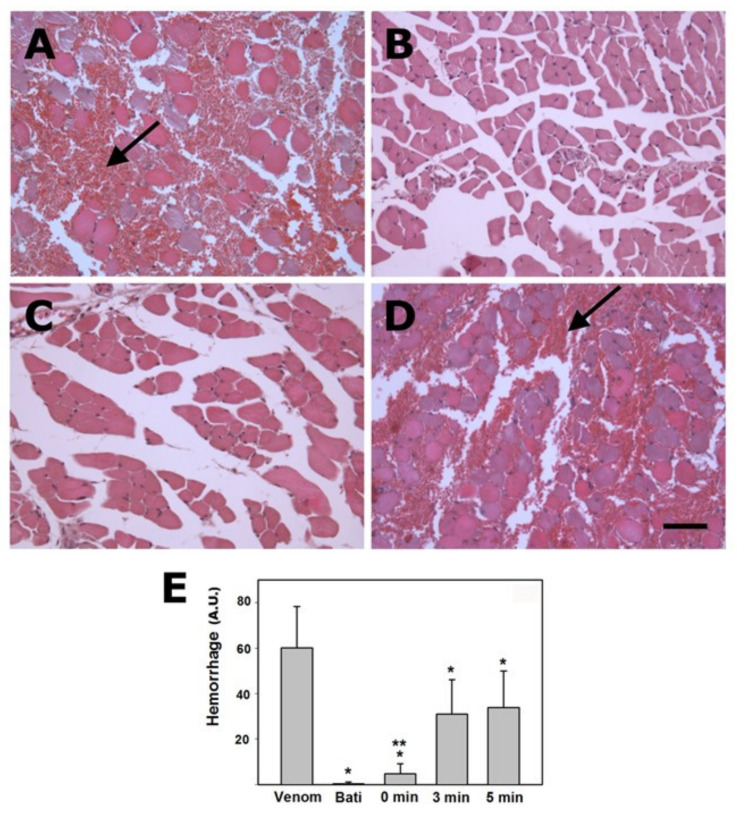
Inhibition by the hydroxamate peptidomimetic Batimastat of the local hemorrhagic activity of the venom of *Echis ocellatus* in muscle tissue of mice by a rescue-type experiment. (**A**) Injection of venom alone (20 µg). Note the abundant local hemorrhage due to the action of SVMPs, as evidenced by masses of erythrocytes (arrow) in the interstitial space. (**B**) Injection of Batimastat alone; no effect is observed in muscle tissue. (**C**) Injection of venom (20 µg), followed immediately by local administration of Batimastat. There is complete inhibition of hemorrhagic activity. (**D**) Injection of venom (20 µg) followed by Batimastat 5 min after envenoming. There are areas of hemorrhage reflecting the rapid action of hemorrhagic toxins. All samples were collected 3 h after venom injection. (**E**) Quantification of hemoglobin in muscle tissue, as an index of hemorrhagic activity. Venom and Bati represent values in mice injected with venom alone (venom) and Batimastat alone (Bati). 0, 3 and 5 min represent values from mice injected with venom and then receiving Batimastat at either 0, 3 or 5 min after envenoming. Results are presented as mean ± S.D. (*n* = 4). * *p* < 0.05 when compared to venom. ** *p* < 0.05 when compared to 3 min and 5 min. Reprinted from Arias et al. (2017), Toxicon 132: 40–49, copyright 2017, with permission from Elsevier.

As previously discussed, in vivo studies for assessing venom inhibition must include not only experiments involving incubation of venoms and inhibitors prior to injection, but also rescue experiments whereby the inhibitors are administered at various time intervals after the injection of venom [119], as has been done in several studies [157,158,159,161] (Figure 6). The route of injection should be carefully considered. In the case of local tissue damage, administration of the inhibitors can be done at the site of venom injection, i.e., intramuscularly or subcutaneously. For practical reasons, the possibility of administering inhibitors by the oral route should be considered. This can be done in the cases of marimastat, DMPS, and the prodrug LY333013, which have good oral bioavailability [159,174]. Since the possibility exists of administering these inhibitors in the field, rapidly after a snakebite, the oral route is important to evaluate. Some inhibitors, like batimastat and LY315920, can be administered by the intravenous route as well, simulating a hospital setting. The inhibitor doses to be selected should be based on the estimation of the corresponding IC_50_ values, determined in preincubation experiments, and based on combined pharmacokinetic and pharmacodynamic evaluations performed in appropriate corresponding models, and/or on doses scaled to those that have been tested in human clinical trials. Experiments may also consider the combined use of inhibitors and antivenoms, hence resembling a situation where inhibitors are administered in the field, or even in the hospital, followed by the normal use of antivenom in a hospital setting [159,174]. This would evaluate whether there are synergistic, additive, or antagonistic interactions between inhibitors and antivenom.

### 7.2. Pharmacokinetic and Biotransformation Issues

Owing to the differences in chemical structure and molecular mass between venom components and synthetic inhibitors, there is likely a mismatch between venom toxicokinetics and inhibitor pharmacokinetics, an issue that must be carefully considered. Moreover, differences in inhibitor pharmacokinetics depending upon the route of injection should be also taken into consideration in the design and interpretation of experiments. There is a possibility that, owing to the effects of venoms on cardiovascular parameters, drug pharmacokinetics may vary in envenomed animals as compared to normal animals, an issue that also needs to be accounted for. Experiments should consider the need to use repeated doses of the inhibitors, owing to pharmacokinetic considerations. This has been shown in the case of LY315920 and LY333013 in a piglet model of envenoming by the coral snake *Micrurus fulvius* [174].

The biotransformation of inhibitors is another issue to consider. This was highlighted in the case of batimastat and marimastat in the experimental envenoming with *Echis ocellatus* venom in mice. In the case of batimastat, the IC_50_ values for the inhibition of local (skin) and systemic (pulmonary) hemorrhage in preincubation experiments, were 0.36 µM and 200 µM, respectively [158]. Since local hemorrhage is tested by injecting the venom-inhibitor mixture into the skin, whereas systemic hemorrhage is assessed by intravenous injection, such discrepancies suggest that, in the circulation, venom SVMPs may dissociate from the inhibitor or that the hydroxamate peptidomimetic drugs are degraded by plasma enzymes, such as esterases, or bind readily to plasma proteins [218].

### 7.3. The Need to Assess Inhibition of Several Toxic Activities

**Figure 7:**
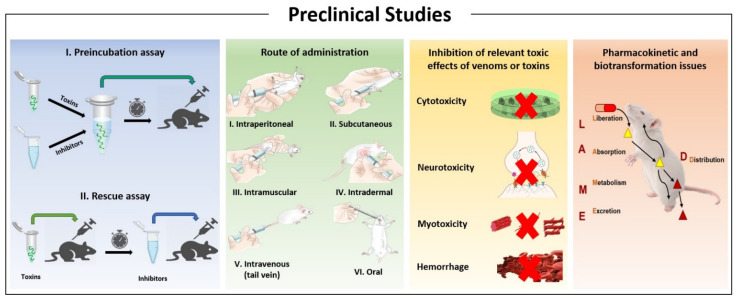
Issues that should be considered when designing experimental protocols to assess the preclinical efficacy of inhibitors against snake venoms or toxins. Experiments must include preincubation and rescue types of assays. The routes of administration of venoms and inhibitors should be chosen by trying to replicate the possible real-life scenario of envenomings. In addition to the study of inhibition of lethal effect of venoms and toxins, the inhibition of other toxicologically relevant activities should be considered, which vary depending on the venom or toxin. Finally, the pharmacokinetic and biotransformation aspects of a particular drug have to be taken into consideration as to adjust the route of injection, the dose, and the need of repeated doses of the inhibitor.

The combined use of SVMP and PLA_2_ inhibitors is highly promising for the treatment of viperid envenomings, owing to the relevance of these enzymes in the overall pathology and pathophysiology of envenomings [159]. Since these envenomings are characterized by a complex series of deleterious effects, a detailed assessment of the potential usefulness of such inhibitory mixtures should consider not only the evaluation of the lethal activity, but also the analysis of inhibition of other relevant effects, i.e., local and systemic hemorrhage, local myonecrosis, in vitro procoagulant activity and in vivo defibrinogenating effect (see for example [157,158]). In some cases, such as with *D. russelii* venom, the inhibition of renal alterations and the systemic capillary leakage syndrome should also be evaluated. Such in-depth assessment provides robust evidence on the preclinical efficacy of the inhibitors to support the design of clinical trials. Figure 7 summarizes some of the main issues to be considered when undertaking the preclinical evaluation of venom inhibitors.

## 8. The Final Goal: Design and Implementation of Clinical Trials for the Evaluation of Inhibitors as Therapeutics for Snakebite Envenoming

The development and preclinical testing of inhibitors for the treatment of snakebite envenomings is a mounting task, but the final challenge has to do with the demonstration of efficacy and safety in clinical trials, an issue that is beyond the goals of this review. Nevertheless, a few issues that need to be considered are next discussed. Few controlled clinical trials on therapies other than antivenoms have been carried out in snakebite envenomings, although recent preclinical developments with repurposed synthetic inhibitors have paved the way for this challenging task, and clinical trials are being planned (Matthew Lewin and Nicholas Casewell, personal communication). Such trials should harness previous experiences with antivenoms, although the use of inhibitors includes some aspects that differ from antivenom studies and may require novel approaches for their design and implementation. For example, it must be decided whether phase I trials should be done or whether previous phase I trials would be acceptable. This is particularly relevant relating to: (i) dose optimization for acute snakebite indication, given that many repurposed drugs will have previously undergone clinical development for chronic disease indications, and (ii) the target population (i.e., predominately people in tropical/sub-tropical low/middle income countries), which may present differences from those who participated in prior phase I trials. Ultimately, all the information from previous trials of repurposed inhibitors should be harnessed to facilitate these new studies.

A relevant issue is the supply of enough synthetic inhibitors manufactured following GMPs. Several aspects on the use of inhibitors in humans should be carefully considered, such as dose, route of administration, time of administration, types of settings where the studies will be carried out (i.e., hospitals or primary health care facilities), consideration on limitations in low income rural settings to carry out these studies, how the administration of inhibitors will be combined with the use of antivenoms, and the follow-up of possible inhibitor side effects or unexpected interactions with antivenoms and other drugs, among other issues. Likewise, the selection of biomarkers and clinical outcomes to assess the efficacy of these new drugs demands careful consideration, yet remains particularly challenging.

In the long term, it is anticipated that snakebite envenoming therapeutics will be strengthened by the combination of: (i) improvement of current antivenoms, both in terms of efficacy and safety, (ii) design of novel and more potent and safer antivenoms, animal-derived or recombinant, (iii) the possible use of inhibitors of clinically relevant toxins, either under field conditions or in hospital settings, (iv) improvements in the accessibility of these therapies in rural settings where most snakebites occur, (v) strengthening of public health systems, including the training of physicians, nurses and other health personnel in the diagnosis and treatment of envenomings, (vi) implementation of follow-up programs for attending the physical and psychological sequelae in affected people, and (vii) improved prevention campaigns and activities based on the involvement of local communities. While aspirational, such a scenario will bring a drastic reduction in the burden of human suffering caused by snakebite, as proposed in the WHO strategy for the control and prevention of these envenomings.

## Data Availability

No new data were created or analyzed in this study. Data sharing is not applicable to this article.
